# Visual search strategies of performance monitoring used in action anticipation of basketball players

**DOI:** 10.1002/brb3.3298

**Published:** 2023-10-24

**Authors:** Yawei Li, Tian Feng, Fuchun Zhang, Umer Asgher, Bingbing Yan, Tianyu Peng

**Affiliations:** ^1^ Department of Sports Physical Education College of Zhengzhou University Zhengzhou China; ^2^ Department of Physical Education Physical Education College of Zhengzhou University Zhengzhou China; ^3^ School of Physical Education Zhengzhou University Zhengzhou China; ^4^ Quality Assurance & NUST International Office Directorate (QA & NIO Dte) National University of Sciences and Technology (NUST) Islamabad Pakistan

**Keywords:** action anticipation, basketball player, eye‐movement, performance monitoring, visual search

## Abstract

**Introduction:**

Numerous studies have found that expert players anticipate better than novices. If more accurate prediction represents performance monitoring of experts, what are the advantages of elite basketball players in identifying and processing available cues? There is still a lack of sufficient evidence. This study examined the visual search in basketball players and explored the performance monitoring of action anticipation, adopting an expert–novice paradigm and eye‐movement technology.

**Methods:**

Forty basketball players were recruited in this study: 20 in the expert group and 20 in the novice group. Participants were asked to predict the outcome of videotaped basketball throws and their accuracy and eye‐movement characteristics were record.

**Results:**

The accuracy of the expert was significantly higher than that of the novice. The experts were able to instantly search and identify important cues in anticipation, and the gaze area of the experts was concentrated on the area of interest of the body. Additionally, the expert group showed long, repetitive, and rapid visual search of vital information, and improved their performance of the task.

**Conclusion:**

The experts could monitor the performance of prediction by grabbing vital shooting information (such as the body of a player). The results suggest the athletes and coaches that if they want to improve the ability of prediction, it may be useful to shift their focus of attention from ball trajectory to body action.

## Graphical abstract

There were 40 participants in this study to predict the outcome of videotaped basketball throws. The accuracy of the expert was significantly higher than that of the non‐athlete and the gaze area of the experts was long, repetitive, and rapid on the area of interest of the body, confirming that professional athletes can conduct performance monitoring during action anticipation.

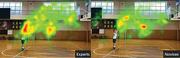

## 1 INTRODUCTION

Anticipation is crucial to expert performance in sport, such as getting a rebound during a basketball game (Williams & Jackson, 2019). Predicting the success or failure of shots can influence the team scores (Gómez et al., 2008). Action anticipation is the ability to predict an event's outcome (Abreu, 2014). Numerous studies have found that expert players anticipate better than novices (Aglioti et al., 2008; Cañal‐Bruland et al., 2015; Li & Feng, 2020; Maglott et al., 2019; Wu et al., 2013). Higher accuracy was found for basketball players when they were predicting their own made shots (Cañal‐Bruland et al., 2015; Maglott et al., 2019). Research conducted by Wu presents that elite athletes can collect information more quickly and effectively in tasks than novices (Wu et al., 2013). Concerning the reason of expert advantage, Williams and Jackson (2019) proposed that they have better interacting perceptual‐cognitive skills, which includes the ability to pick up postural cues, identify familiarity in evolving sequences of play, and to use the available contextual information to facilitate anticipation.

During a fierce competition, basketball players need to process abundant information to complete challenges. To make accurate anticipation, they may lie not only in the flight trajectory of the ball but also in the body cues involved in the prediction process (Miller & Bartlett, 1993). By monitoring the eye‐movement patterns, research can identify and evaluate the decisive visual cues when an athlete was scanning the environment and making predictions (Aglioti et al., 2008; de Castro Ribeiro et al., 2021; Khalaji et al., 2021). Aglioti et al. (2008) found that basketball players can obtain important information through their visual receptors, and then recognize movements and spatial position in the game. Accordingly, the experts’ visual system is used more effectively to pick up vital information by integrating the use of peripheral vision and the fovea (Williams & Jackson, 2019). However, successful and failed free throws conduct different kinematic information of the shooting player's body (Mullineaux & Uhl, 2010), and knee, wrist, and finger joint angles are significant cues for accurate predictions. In particular, Uchida et al. (2014) compared the eye‐movements of experienced players and novices when they are watching a basketball free throw video. Their result showed that experienced players had significantly higher accuracy and gazed more at the lower part of the player's body than the novices.

Predicting the fate of a basketball often needs fast and correct reaction, and a together work of brain resources, vision, attention, and decision‐making. Unfortunately, excellent players make wrong judgments. Given this, after a prediction error occurs, whether the athletes could identify and monitor their own mistakes, and then adjust and correct their errors, or they are unable to detect their mistakes and even neglect mistakes? Li and Feng (2020) proposed that when the prediction is inconsistent with the actual result, whether athletes can detect mistakes in time and effectively correct strategy is determined by their level of performance monitoring. Therefore, performance monitoring refers to discovering and correcting the difference between the response that should be made and the actual response (Li & Zhang, 2015). Scheffers and Coles suggested that the level of error awareness is related to the evaluation of behavioral correctness (Scheffers & Coles, 2000). A functional magnetic resonance imaging (fMRI) study found that basketball athletes had greater activation in the insula (an area related to alertness) when they make an incorrect judgment on the outcome of shots, suggesting that they may have the ability to detect wrong judgments (Wu et al., 2013). Moreover, some researchers demonstrated that active inference, which proposes that perception and action are underpinned by the organism's need to remain within certain stable states, can account for relevant perception and action prediction, such as error minimization and precision‐weighted inference (Harris et al., 2022).

However, if more accurate prediction represents performance monitoring of experts (Li & Zhang, 2015; Li & Feng, 2020), what are the advantages of elite basketball players in identifying and processing available cues? There is still a lack of sufficient evidence. This study takes basketball players in different sports levels as subjects, combines the expert–novice paradigm and eye‐movement technology, and uses a basketball shooting video as stimulus material to study the strategies of basketball players’ visual search, as well as the cue process of performance monitoring. It was hypothesized that (1) the expert group will show better accuracy of prediction than novices and (2) the gaze area of expert is concentrated in the body, showing longer gazing time and more fixation frequency.

## 2 METHODS

### 2.1 Participants

Forty participants were included in this study: 20 in the expert group and 20 in the novice group. The expert group consisted of collegiate basketball players, with an average age of 20.55 ± 1.25 years, an average training period of 8.86 ± 4.16 years, and the weekly average training time of 9.11 ± 2.90 h. The novice group consisted of college students majoring in physical education but not in basketball who had taken a single semester of basketball general courses. There was no significant difference in the average age of the two groups of participants (*p* = .71). Power analysis was conducted with G*Power software, using the setting for expected effects size at 0.25, *α*‐level at .05, sample size at 40, and the power (1 − *β*) was 0.96. The participants were all right‐handed and in good health. They all volunteered to participate in the experiment and signed an informed consent form after being informed of the content and procedures of the experiment. They were given a small amount of remuneration after the experiment.

### 2.2 Materials

The stimulus material for the experiment was a basketball free throw video of two professional right‐handed, male collegiate basketball players (who were experts and did not participate in the experiment). The video is a high‐definition video in MP4 format with a size of 1088 pixels × 608 pixels and 60 frames per second. During the video shooting, two athletes were asked to practice the standard free throw, and the camera was set up at a height of 1.70 m. Each video is about 1500 ms (90 frames in total), which starts with the player's preparation of holding the ball and ends after making or missing a shot.

After consulting professional basketball coaches and athletes, we selected 40 shooting scenes. Half of them are successful shots and half are missed shots (but also hit the basket or backboard). Each shot was about 1200 ms, started from the preparation of the shot to the ball flies to the midpoint of the top of the arc and the basket. To prevent participants from watching the shooting results, we deleted the end of the video. The video was edited using Adobe Premiere Pro software (Adobe Systems), and the experimental design and presentation were completed using E‐prime 2.0 (Psychology Software Tools).

### 2.3 Procedure

**FIGURE 1:**
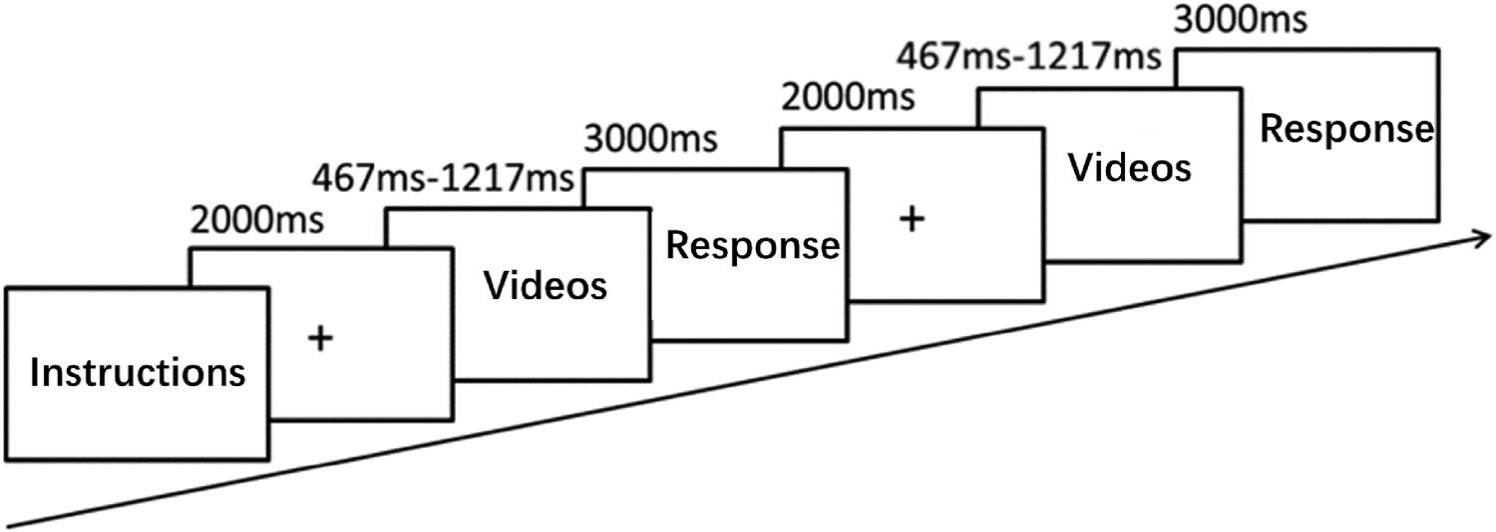
The procedure of the experiment.

The experiment was completed in the laboratory of the Physical Education College of Zhengzhou University. The participants sat in front of a 23.8‐inch computer screen with a refresh rate of 100 Hz and a resolution of 1920 × 1080. The participants’ eyes were kept level with the screen at 60 cm. In this study, Tobii Pro Glasses 2 eye tracker (binocular, 100 Hz) was used to automatically record the eye‐movement characteristics of the participants in the experiment processing visual information in real time. The glasses have a scene camera that could take 1920 ×1080 video at 25 fps and the field of view of scene camera 90° with four eye tracking sensors. To be able to collect accurate eye tracking data, the gaze of each participant was calibrated individually by looking at and focus on the center of the one‐point calibration card, which was held flat against a wall. The distance between the participant and the calibration card was 1.0 m. Participants were asked to predict the outcome of videotaped basketball shootings. First, a gaze point of 2000 ms appeared on the screen to allow the participants to focus their attention, and then a 467–1217 ms basketball free throw video was played. The video disappeared after playing, and then the response screen appeared. The participants needed to quickly and accurately judge whether the shot they just saw would be successful within 3000 ms (“F” key and “J” key represented either “hit” or “miss,” and were counterbalanced between subjects). A timeout was judged as an error. Then the next trial began. The experimental process has been used by many researches (Aglioti et al., 2008; Cañal‐Bruland et al., 2015; Wu et al., 2013) and is shown in Figure 1. Participants were required to complete 15 practice trials with feedback. The practice videos were taken along with the formal videos, but were different from the formal ones. An accuracy rate of over 60% was considered a passing result, and the formal experiment would then begin. The formal experiment consisted of 40 (video clips) × 4 (judgment time points) × 2 (repetitions) = 320 trials, divided into eight blocks, and each block consisted of 20 trials with the same judgment time points. The entire experiment was monitored by a lead examiner.

### 2.4 Data recording and analysis

**FIGURE 2:**
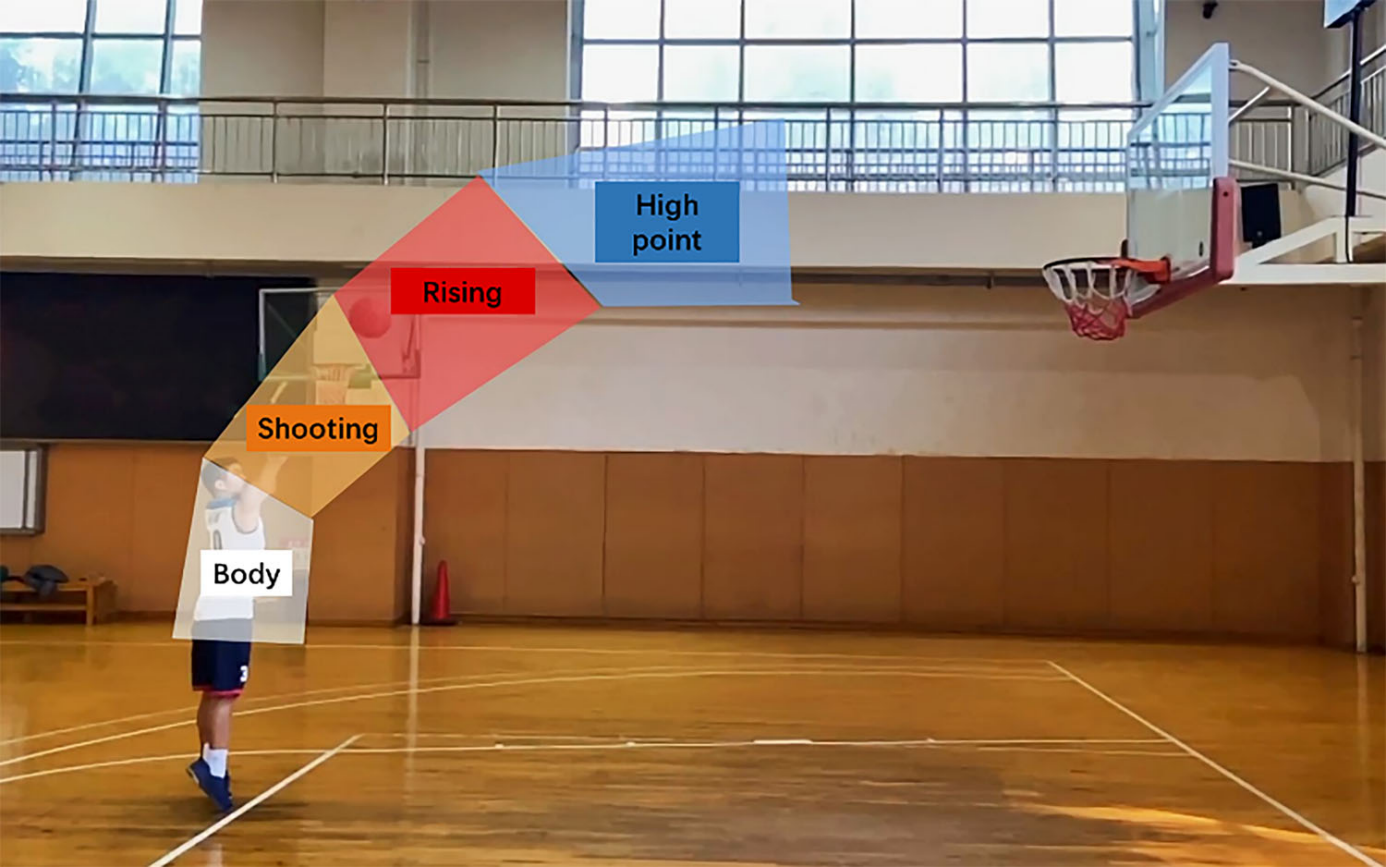
Division of the areas of interest during a free‐throw shooting from the beginning of body movement to the high point of ball trajectory. There are four regions for eye‐movement analysis: (1) the player's body, (2) ball shooting, (3) ball rising, and (4) the high point.

For the behavioral data, only accuracy was analyzed because the participants were only allowed to press the button after the picture disappeared, and it would have been difficult to accurately observe the reaction time, similar to previous research (Zhao et al., 2018). Participants whose accuracy was lower than 85% (one athlete and one novice) were excluded. Normal distributions for the accuracies in two groups were confirmed (*z* < 0.131, *p* > .196 in all instances). Accuracy was analyzed using an independent *t*‐test, and the between‐group factors were groups (expert group, novice group). Eye‐movement data were collected with the Tobii X60 eye‐movement device. As shown in Figure 2, the gaze target was divided into four areas of interest (AOI) according to previous studies (Wu et al., 2013): the player's body, ball shooting, ball rising, and the high point. The dependent variables for the eye tracking were first fixation duration, average fixation duration, average number of fixations, locus of fixation, peak velocity, and heat map. Fixation duration is the sum of fixation times for each fixation area, which reflects the speed and efficiency of the subject's processing of the stimulus material. Fixation number refers to the fixation frequency per unit time, which can be used as a measurement index of fixation stability. Locus of fixation, which are the lines of fixation areas, can reflect the gaze patterns of basketball players and novices. A repeated‐measures analysis of variance was performed for the above eye‐movement indicators, the between‐group factors were the groups, and the intra‐group factors were the areas of interest. The Bonferroni method was used to conduct post hoc testing.

## 3 RESULTS

### 3.1 Behavioral results

To compare the accuracy of the two groups, *t*‐tests were performed. As shown in Table 1, the accuracy of the expert (0.71 ± 0.09) was significantly higher than that of the novice (0.38 ± 0.02), *t* (36) = 2.503, *p* < .001, *d* = 5.062. The results indicate that the expert predicts the accuracy of action better than the novice.

**TABLE 1:** Accuracy of expert and novice groups.

Group	Number of participants	*M* ± SD
Experts	20	0.71 ± 0.009
Novices	20	0.38 ± 0.002

Abbreviations: *M*, mean; SD, standard deviation.

### 3.2 Eye‐movement results

#### 3.2.1 First fixation duration

**FIGURE 3:**
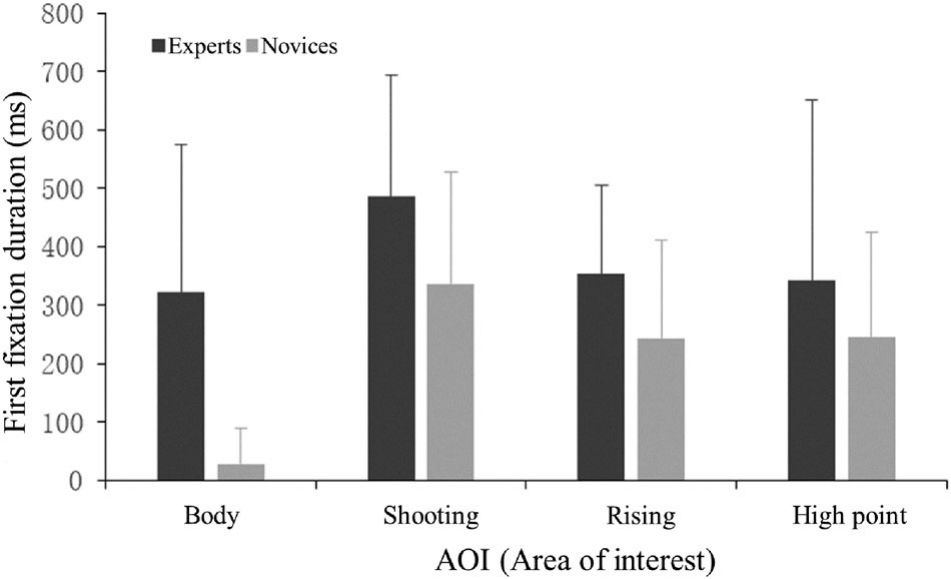
Comparison of the duration of the first fixation between the experts and novices.

As shown in Figure 3, the results of the duration of the first gaze at the AOI showed that the main effect of AOI was significant, *F*(3, 108) = 6.74, *p* < .001, η_p_^2^ = 0.272. Post hoc test found that the duration of the first gaze at the AOI was significantly shorter than those of shots and high points (all *p* < .012). The main effect of the group was significant, *F*(1,36) = 6.59, *p* = .019, η_p_^2^ = 0.268, and the post hoc test demonstrated a longer duration of the first gaze at the AOI for the expert (375.95 ± 44.75 ms) than the novice (213.50 ± 44.75 ms).

#### 3.2.2 Average fixation duration

**FIGURE 4:**
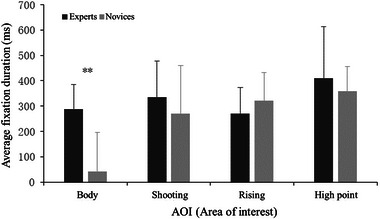
Average fixation time for the area of interest of the experts and novices.

The main effect of AOI was significant (Figure 4), *F*(3, 108) = 9.92, *p* = .000, *η*_p_^2^ = 0.335. Post hoc tests found that the average fixation duration of the body (164.90 ± 35.59 ms) was significantly shorter than that of the hand (385.00 ± 24.01 ms) and high point (302.15 ± 28.75 ms). A marginally significant main effect was found for experts, *F*(1,36) = 3.89, *p* = .064, *η*_p_^2^ = 0.178, indicating that the expert had a longer average fixation time for the AOI than the novice. In addition, the interaction between them was significant, *F*(3, 108) = 4.56, *p* = .006, *η*_p_^2^ = 0.202. Post hoc tests found that the average fixation time of the expert was significantly longer than that of the novice (*p* = .003).

#### 3.2.3 Average number of fixations

**FIGURE 5:**
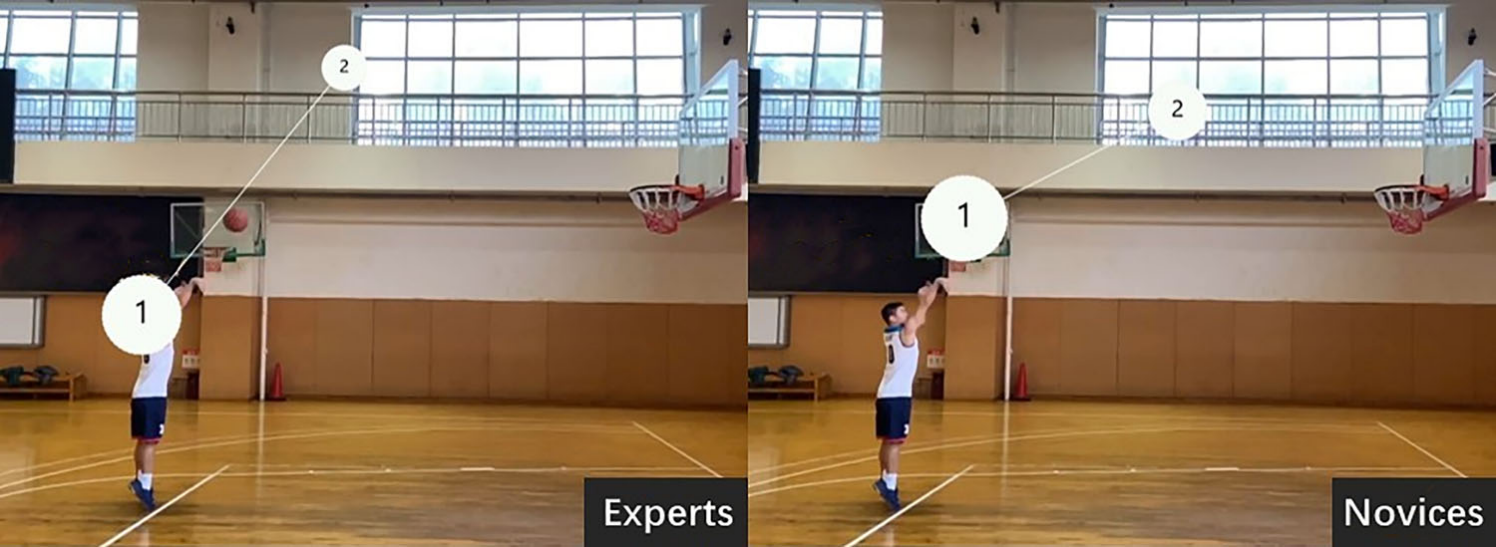
Fixation times for the area of interest in the experts and novices.

The main effect of AOI was significant (Figure 5), *F*(3, 108) = 5.97, *p* = .006, *η*_p_^2^ = 0.249. Post hoc tests found that the fixations number of the body was significantly lower than that of shooting, rising, and high point (all *p* < .010). The main effect of the group was not significant, *F*(1,36) = 1.47, *p* = .241, *η*_p_^2^ = 0.076, but the data revealed that the fixation numbers for shooting in the expert (24.50 ± 5.95) tended to be longer than those in the novice (12.95 ± 5.95); expert fixation times for the body (6.20 ± 1.48) were longer than those of the novice (0.80 ± 1.48). Besides, 80% of the novice did not look at the body during the test. The interaction between the two variables was not significant, *F*(3, 108) = 1.10, *p* = .343, *η*_p_^2^ = 0.058.

#### 3.2.4 Locus of fixation

**FIGURE 6:**
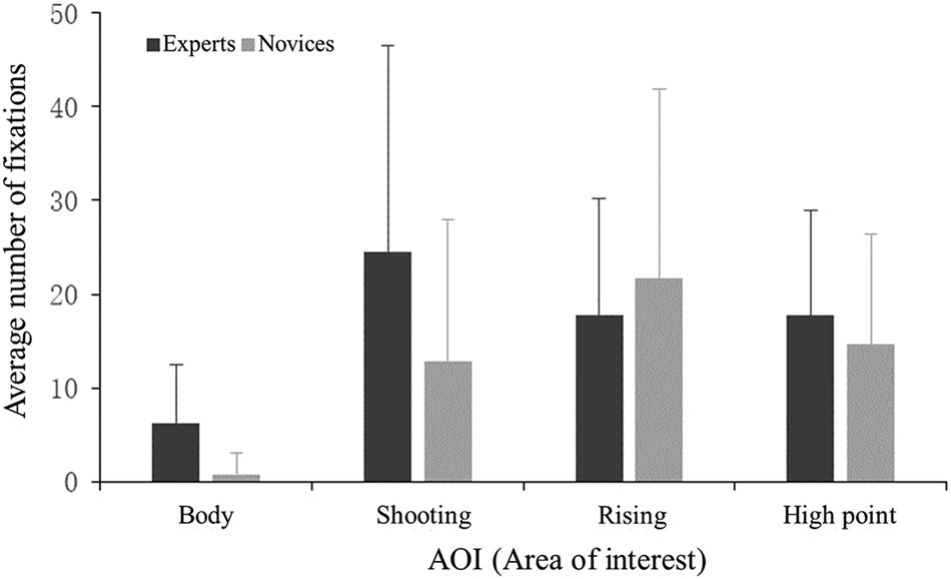
Sequences of the experts and novices looking at the area of interest for the first time.

The locus of fixation refers to the trajectory of fixation. The results of the gaze at AOI showed that the main effect of AOI was significant, *F*(3,108) = 7.82, *p* < .001, η_p_^2^ = 0.303. Post hoc tests showed the time to look at the body for the first time was significantly earlier than the ball shooting and the high point (all *p* < .031). In addition, the interaction effect between the two was significant, *F*(3, 108) = 3.30, *p* = .048, η_p_^2^ = 0.155. The experts’ first gaze time for the first AOI was significantly earlier than that of the novice (*p* = .021). As shown in Figure 6, the search process of each group of participants can be seen from the sequence of cues when they first gaze at the AOI. The expert was body→high point; the novice was rise→high point.

#### 3.2.5 Peak velocity

**FIGURE 7:**
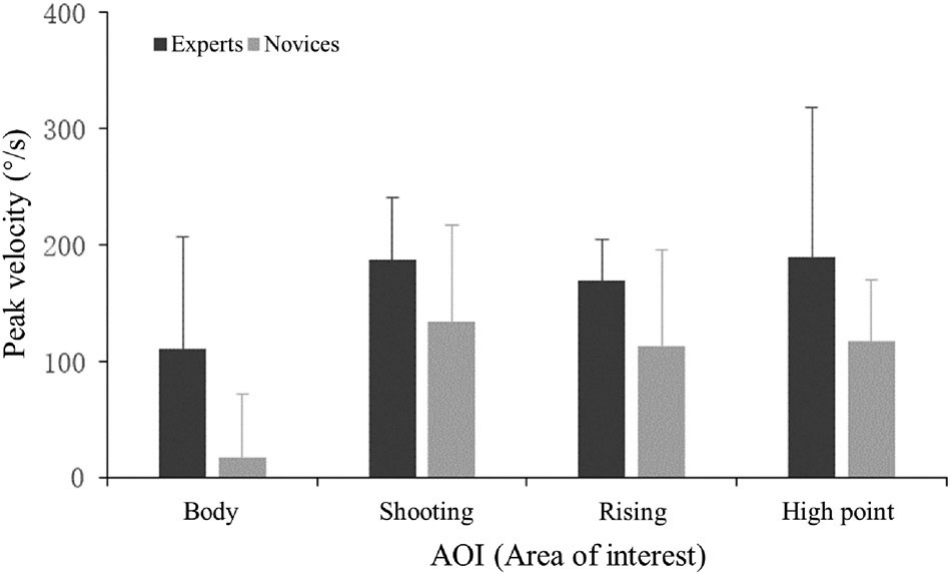
Peak angular velocity of the area of interest in the experts and novices.

The results indicated that the main effect of the AOI was significant (Figure 7), *F*(3, 108) = 7.62, *p* = .002, *η*_p_^2^ = 0.279. Post hoc tests showed that the speed of saccade in the AOI of the body was significantly shorter than that of shooting, rise, and high point (all *p* < .012). The main effect of group was significant, *F*(1,36) = 10.10, *p* = .005, *η*_p_^2^ = 0.359. The highest peak of saccade velocity in the expert (163.80 ± 15.22 mm/s) in the AOI was greater than that of the novice (95.42 ± 15.22 mm/s).

#### 3.2.6 Heat map

**FIGURE 8:**
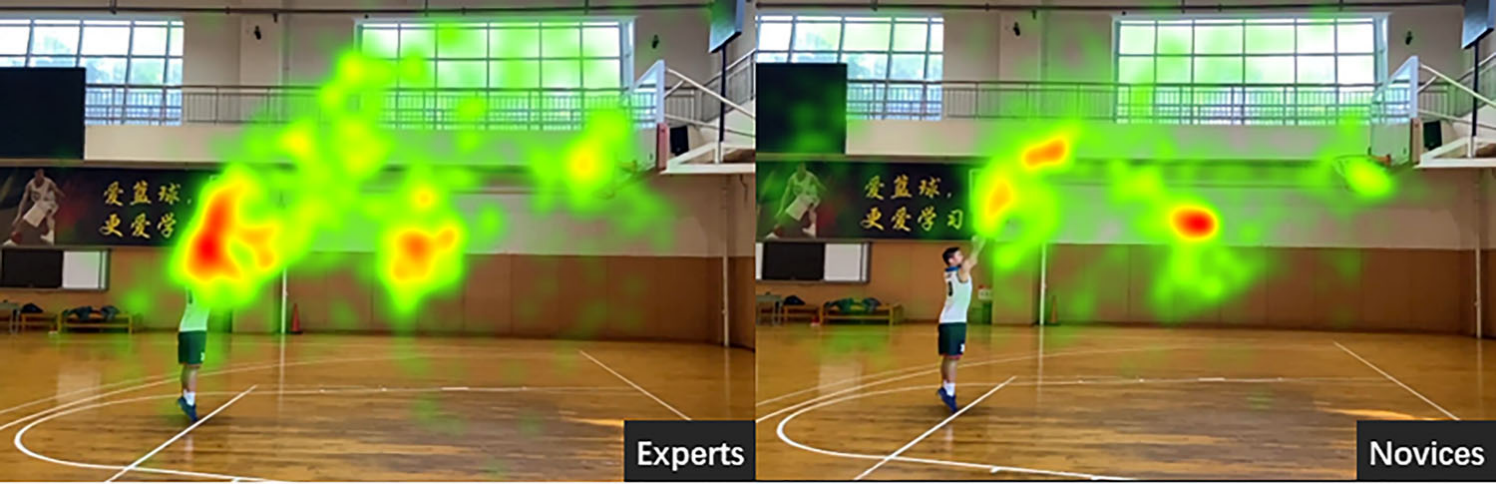
Eye‐movement heat maps of the experts and novices.

As shown in Figure 8, the fixation results of the two groups of participants are significantly different. The expert focused on the body and arms in the picture. For the panel of experts, the red area of the body is darker and larger than the red area of the shooting. The focus area of the novice group's gaze is at the stage of the shooting and the ball rising. The novice has a larger red area in the rising area of the ball than the shooting area. There is a significant difference in the degree of gaze between the two groups. The focus area of the expert is larger, while the novices’ is smaller, which focuses on the shooting and rising area of the ball.

## 4 DISCUSSION

The present study adopted an expert–novice paradigm and eye‐movement technology to explore the strategy of the visual search in basketball players at various sports levels and explored the ability of performance monitoring in action anticipation. The results showed that the accuracy of the expert was significantly higher than that of the novice, indicating that the skilled players could conduct more precise predictions. Additionally, the expert showed long‐term, repetitive, and rapid visual searching. In other words, the experts could monitor the performance of prediction by grabbing vital shooting information (such as the body of a player).

First of all, the behavioral results confirmed the advantage of predicting the ball fate for the experts. Similar to previous studies (Aglioti et al., 2008; Cañal‐Bruland et al., 2015; Li & Feng, 2020; Maglott et al., 2019; Wu et al., 2013), they showed higher accuracy for both successful and missed balls than the novices. However, if more accurate prediction represents performance monitoring of experts, what are the advantages of elite basketball players in identifying and processing available cues? Moreover, after a prediction error occurs, how do athletes identify, monitor, adjust, and correct their errors by collecting relevant information? Recording the eye‐movement of experts and novices could answer the questions.

The number and duration of fixations have been used by a large number of studies to reflect the temporal characteristics of the visual search of athletes (Aglioti et al., 2008; de Castro Ribeiro et al., 2021; Khalaji et al., 2021; Savelsbergh et al., 2002). These findings are consistent with the results of this study. The average time and number of fixations in the AOI of the expert athlete were significantly greater than those of the novice, which showed that the expert spent more time on the essential information and examined that region more frequently. Studies have shown that experienced basketball players can predict whether the ball will go through the hoop by observing the angle variables of the shooter's lower body and arm, wrist, and finger joints (Yukimasa et al., 2010). In this study, through the heat map of eye‐movement for two groups, it was found that the focus area of the expert was the player's body and arm. In this area, the fixation area was larger and more concentrated, and the red area of the body area was more intense and larger than the red area of shooting in the expert. Uchida et al. (2014) compared the eye‐movements of novice and experienced basketball players while observing basketball free throws and found players tend to focus more on the lower part of their body than novices when watching a video at normal speed. A study found that by integrating the use of peripheral vision and the fovea, the expert's visual system is used more effectively to obtain important information (Williams & Jackson, 2019). Thus, experts can optimize information processing by selectively allocating attention resources to task‐related stimuli and ignoring irrelevant ones (Brams et al., 2019).

Athletes can selectively allocate attention to important information relevant to the task through effective gaze and gain more time to focus on the key areas (Brams et al., 2019). In this study, the superior performance of experts was proved and the heat map showed that the expert could search and recognize important cues more accurately than the novice. The duration of the first fixation on the AOI of the expert was significantly longer than that of the novice. In this regard, the expert paid attention to the body AOI for a long time. Zwierko et al. (2018) found that shooting accuracy was positively influenced by longer fixations and less frequent. Toh et al. (2021) found that the target location sequence learning could be disrupted by variability in distractor locations, revealing that the experts may have a better ability to extract and use spatiotemporal regularities in complex environments through years of training. Therefore, the results of the peak velocity of saccade in the AOI showed that the expert was faster than the novice, indicating that the expert could search and monitor effective information through rapid saccade performance in the AOI. Additionally, comparing the fixation locus of experts and novices, it was presented that the visual search of the expert is from body to the high point, while the novice is from the ball rising to high point. That is to say, the expert first gazed at the body area before the shooting of the ball to identify and predict the shooting, and the novice gazed at the trajectory of the rising ball after shooting. This kind of advantage may be a sensorimotor resonance. A study proposed that skilled athletes rely preferentially on sensorimotor resonance mechanisms that simulate the actions of others in their motor systems (Hiroki et al., 2021).

The current study pays close attention to the visual search strategies of basketball experts and novices and provides evidence for how basketball experts conduct the performance monitoring of their anticipation. The results suggest the athletes and coaches that if they want to improve the ability of prediction, it may be useful to shift their focus of attention from ball trajectory to body action. The present study has two limitations. First, as the eye‐movement device in the present experiment only allows block‐designed task, it is difficult to compare the attentional focus of successful and missed shots. Future studies should focus on this interesting topic. Second, studies using the first view of action anticipate often reported the subject’ arm or basketball may block the view and thus affected the judgment (de Oliveira et al., 2008; Vickers et al., 2019). The angle of view of the video in this study was the lateral view of a basketball shooting. This view is closer to watching someone else's shot (as is the case in most basketball games), but it is quite different from the view of one's own shot.

## 5 CONCLUSION

The expert can search and identify important cues during the prediction process, focus on the body area in the areas of interest, and invest more cognitive resources. The long‐term, repeated, and rapid visual search and monitoring of key information of the expert of the AOI can affect the performance and results of the predicted task. The results showed that the experts’ accuracy of judgment was significantly higher than that of the novice. The expert is focused on the body area in the AOI and invests more cognitive resources.

## AUTHOR CONTRIBUTIONS

**Yawei Li**: Conceptualization; data curation; investigation; supervision; writing—original draft. **Tian Feng**: Conceptualization; data curation; formal analysis; funding acquisition; investigation; writing—review and editing. **Fuchun Zhang**: Data curation; investigation; resources; software; visualization. **Umer Asgher**: Formal analysis; resources; supervision. **Bingbing Yan**: Investigation; visualization. **Tianyu Peng**: Investigation; software.

## CONFLICT OF INTEREST STATEMENT

The authors declare no conflicts of interest.

### PEER REVIEW

The peer review history for this article is available at https://publons.com/publon/10.1002/brb3.3298

## Data Availability

The data that supports the findings of this study are available in the Supporting Information of this article.
